# Nutrient Status Assessment in Individuals and Populations for Healthy Aging—Statement from an Expert Workshop

**DOI:** 10.3390/nu7125547

**Published:** 2015-12-16

**Authors:** Szabolcs Péter, Wim H. M. Saris, John C. Mathers, Edith Feskens, Annemie Schols, Gerjan Navis, Folkert Kuipers, Peter Weber, Manfred Eggersdorfer

**Affiliations:** 1 Nutrition Science & Advocacy, DSM Nutritional Products Ltd., Wurmisweg 576, Kaiseraugst 4303, Switzerland; peter.weber@dsm.com (P.W.); manfred.eggersdorfer@dsm.com (M.E.); 2 NUTRIM School of Nutrition and Translational Research in Metabolism, Maastricht University Medical Centre, Universiteitssingel 40, Maastricht 6229 ER, The Netherlands; w.saris@maastrichtuniversity.nl (W.H.M.S.); a.schols@maastrichtuniversity.nl (A.S.); 3 Human Nutrition Research Centre, Institute of Cellular Medicine, Campus for Aging and Vitality, Newcastle University, Newcastle upon Tyne NE4 5PL, UK; john.mathers@newcastle.ac.uk; 4 Division of Human Nutrition, Wageningen University, Bomenweg 4, Wageningen 6703 HD, The Netherlands; edith.feskens@wur.nl; 5 University Medical Center Groningen, University of Groningen, Hanzeplein 1, Groningen 9700 RB, The Netherlands; g.j.navis@umcg.nl (G.N.); f.kuipers@umcg.nl (F.K.); 6 University Hohenheim, Schloß Hohenheim 1, Stuttgart 70599, Germany

**Keywords:** nutrient, status, aging, patients

## Abstract

A workshop organized by the University Medical Center Groningen addressed various current issues regarding nutrient status of individuals and populations, tools and strategies for its assessment, and opportunities to intervene. The importance of nutrient deficiencies and information on nutrient status for health has been illustrated, in particular for elderly and specific patient groups. The nutrient profile of individuals can be connected to phenotypes, like hypertension or obesity, as well as to socio-economic data. This approach provides information on the relationship between nutrition (nutrient intake and status) and health outcomes and, for instance, allows us to use the findings to communicate and advocate a healthy lifestyle. Nutrition is complex: a broader profile of nutrients should be considered rather than focusing solely on a single nutrient. Evaluating food patterns instead of intake of individual nutrients provides better insight into relationships between nutrition and health and disease. This approach would allow us to provide feedback to individuals about their status and ways to improve their nutritional habits. In addition, it would provide tools for scientists and health authorities to update and develop public health recommendations.

## 1. Introduction

Throughout a lifespan, inadequate nutrition is related to several chronic diseases that greatly impact morbidity, mortality, and quality of life [1]. Adequate nutrition is essential to a healthy life and healthy aging on an individual as well as on a societal level. However, there is moderate awareness about this issue worldwide and communities must also overcome the hurdle from awareness to action. The important role that nutrients play in supporting healthy aging and preventing non-communicable diseases is currently receiving insufficient attention and remains poorly understood not only in the general population, but also among policymakers and health professionals. Insights into the nutrient status of individuals and populations and understanding of the impact of optimizing nutrient status during a lifespan in different life conditions is unsatisfactory. The public health implications of balancing nutrient intakes and thus reducing malnutrition and the prevalence of non-communicable disease are enormous, consequently cutting down healthcare spending around the world [2].

Malnutrition can be avoided by improving diets in addition to continuous nutritional education by simple and cost-effective measures such as special food supplements and nutrient supplementation of vulnerable populations or food fortification with micronutrients for the general population. Addressing the problem of malnutrition should not only be focused on dietary factors but also on the multiple risk factors that underlie malnutrition, including physical, social, and medical factors. Nutrition and diet should be considered together with other socio-economic issues as an integral part of the solution to achieve nutrient adequacy and support healthy aging. Maximizing the number of healthy life years by addressing inadequate nutrient status to tackle malnutrition in the general population and in specific risk groups is an investment that will pay off but requires a comprehensive national action plan in each country.

Considering the importance of essential micronutrients for a healthy life and in healthy aging, on 24 March 2015 the University Medical Center Groningen convened a group of scientists and clinicians to discuss the role of micronutrient status in healthy aging, and the importance of nutrient status assessment in individuals and populations. This paper summarizes the main discussion points and conclusions of this symposium.

## 2. Assessment of Nutritional Status

**Figure 1:**
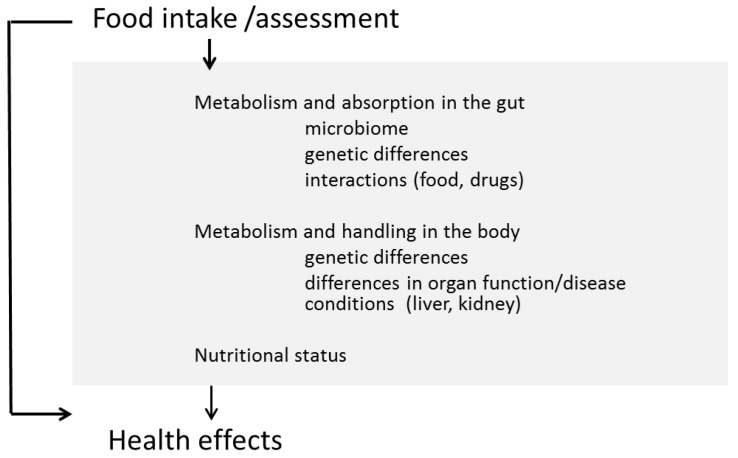
Assessment of nutritional status offers the potential to improve health outcomes.

Knowing the current micronutrient status is an important pre-requisite while developing concepts to improve nutrition in general. Food intake surveys are widely used to assess nutritional status and are available for many countries. In many cases they indicate that people do not manage to achieve a balanced diet and intake of essential nutrients according recommendations [3]: for example, in Germany, 40% of the people do not eat vegetables regularly and 70% do not eat the recommended amount of fruits. As a consequence, intake of essential nutrients is below recommendations [4]. However, intake surveys are often challenged due to the well-recognized considerable risk of under- and/or over-reporting (Table 1) [5,6]. Surveys also do not reflect the actual situation in an individual because of variable factors like bioavailability of the various nutrients and the impact of genetic makeup and other factors that are not taken into account (Figure 1). Nutritional status by using valid biomarkers measured in biological samples like blood or urine or other technologies may provide more accurate data as they more accurately reflect the actual situation in humans. Vitamin D is an example of a micronutrient for which representative data are available in many countries for the general population as well as for risk groups like pregnant women, children, and the elderly [7,8]. For vitamin D reference values for optimal status are defined and broadly used by medical doctors and health care professionals [9,10].

**Table 1:** Limitations of current strategies to assess nutrient intake [6].

Nutrient Intake Assessment Method	Limitation
*24-h recall*	One record is seldom representative of a person’s usual intake
	Under-reporting/over-reporting occurs
	Relies on memory
	Omissions of dressings, sauces, and beverages can lead to low estimate of energy intake
	Data entry can be very labor intensive
*Food record*	Requires high degree of cooperation
	Response burden can result in low response rates when used in large national surveys
	Subject must be literate
	Takes more time to obtain data
	Act of recording may alter diet
	Analysis is labor intensive and expensive
*Food frequency questionnaires*	May not represent usual foods or portion sizes chosen by respondents
	Intake data can be compromised when multiple foods are grouped within single listings
	Depend on ability of subject to describe diet
*Diet history*	Lengthy interview process
	Requires highly trained interviewers
	Difficult and expensive to code
	May tend to overestimate nutrient intake
	Requires cooperative respondent with ability to recall usual diet
*Duplicate food collection*	Expense and effort of preparing more food
	Effort and time to collect duplicate samples
	May underestimate usual intake
*Food account*	Does not account for food losses
	Respondent literacy and cooperation necessary
	Not appropriate for measuring individual food consumption
*Food balance sheet*	Accuracy of data may be questionable
	Only represents food available for consumption
	Does not represent food actually consumed
	Does not account for wasted food
*Telephone interviews*	Subject to many of the same disadvantages of collecting 24-h recall and food record data
	Estimating portion sizes in recalls may be difficult
*Photograph and video*	Periodic revalidations are recommended
	Unable to distinguish visually similar foods or document preparation methods
	Subject to technical problems

## 3. Improvement of Individual Nutritional Needs

Over the last century, nutritionists have worked intensively on the question how to assess, and address, the nutritional needs of individuals and groups. Many reports have been published on the Recommended Daily Allowances (RDA) of macro- and micronutrients [11,12]. Upon evaluating all these efforts, one has to conclude that, so far, the scientific outcome is not satisfactory and difficult to understand by the general public due to the ongoing debate between scientists about the relation between adequate intake of a given nutrient and health [13]. Lack of validated biomarkers for nutrient intake and the discussed inherent problem of unreliable food intake data has hampered the nutritional field enormously [14,15,16].

Furthermore, it can be questioned whether recommendations for the population at large are specific enough to address the individual needs. Also, the individual consumer nowadays does not necessarily accept and follow general recommendations. Most probably a more personalized nutritional approach could help to motivate the individual consumer to change to a healthier diet and lifestyle (Table 2). The concept of personalized nutrition emerged following the sequencing of the human genome in 2000. It was hoped that with the identification of gene-nutrient interactions, an individual’s response to particular diets would be better understood and therefore appropriate dietary modifications could be made to optimize health and lower disease risk [17]. Although research in the area of nutrigenomics has deepened our insight into the relationship between nutrition and the activity of specific genes, the translation of this knowledge to sound public health advice has not yet been reached. In addition, early attempts to produce a sustainable business model through personalized nutrition services have been unsuccessful leading to a sceptical vision for the future. Despite this, the potential of nutrigenomics in advancing public health awareness and delivery is too great to be dismissed without further exploration. This has led to the European Union (EU) Food4Me project which aims to provide answers and new technologies to shape our thinking about personalised nutrition and extend the current state-of-the-art by: (1) developing new scientific tools for the exploration of dietary, phenotypic and genotypic data in the delivery of personalized nutrition using an internet approach; (2) assessing the validity of delivering a personalised nutrition service in a Proof of Principal (PoP) research study, involving a large cohort of 1280 volunteers across seven EU states [18]. Results of the PoP study showed that an internet-based personalised nutrition intervention was effective in improving dietary behaviors compared with conventional population-based advice [19].

**Table 2:** Modern paradigm of nutritional sciences focuses on dietary patterns and nutritional status (Institute of Medicine, USA, 2006).

	Classical	Modern
*Basis for RDA*	Prevention of disease	Optimal health
*RDA designed for*	Groups	Groups and individuals
*Type of evidence*	Clinical “depletion-repletion” model	Metal-analysis of RCTs
		Nutrigenomics
		System biology approach

RDA: Recommended Daily Allowances; RCT: Randomized Controlled Trial.

## 4. Role of Nutritional Status in Healthy Aging

**Figure 2:**
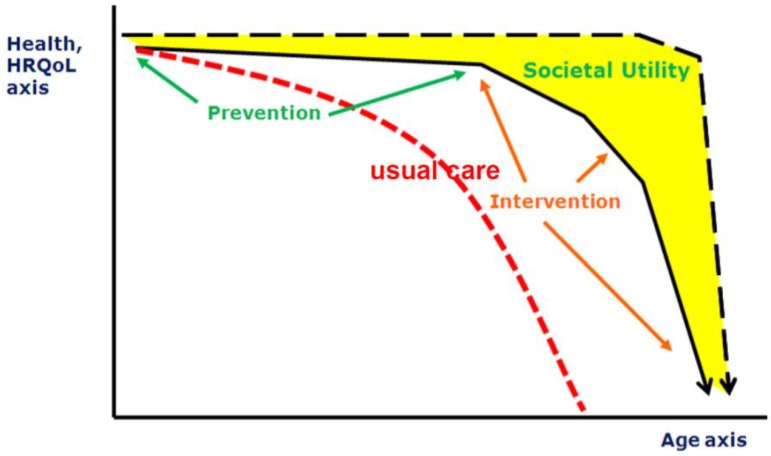
Different scenarios of aging (HRQOL: Health-Related Quality of Life).

The combination of reduced birth-rates and increased longevity results in a major shift in the age distribution of populations towards larger proportions of older people. The population in Europe is aging rapidly: its median age is already the highest in the world, and the proportion of people aged 65 and older is forecast to increase from 14% in 2010 to 25% in 2050 [20]. However, in particular, the last part of the elongated life span is complicated by diseases which are not only is a burden for the individual but also will be a “grand challenge” for public health care systems. Therefore, finding ways to reduce risks of illness and frailty in the elderly is one of the goals defined by the European Union [21]. The factors which can contribute to a healthy life and life span are manifold, nutrition being one of them. Whilst it is accepted that nutrition is a key modulator of health across one’s life-course, there is considerable ignorance about the most effective ways of changing dietary behaviors of younger and older people and about the outcome measures that will be most informative [22]. These research gaps limit opportunities to improve the nutritional status of people in all stages of their lives and hence to enhance healthy aging [23]. This starts even before birth: through nutritional status of the (grand) parents, nutrition steers the health status of the offspring by various (e.g., epigenetic) modes of action. After birth, nutrition modifies health status throughout all stages of the lifespan, and hence has a critical role in primary prevention (preventing onset of disease), secondary prevention (preventing progression of disease), and tertiary prevention (maintenance of function; Figure 2). Moreover, high risk groups (e.g., pregnant women, infants, elderly, patients) have specific (and different) nutritional needs.

It appears that there is a wide discrepancy between outcomes of epidemiological association studies supporting the potential of nutritional interventions and the disappointing outcomes of many nutritional intervention studies on health status. Resolution of this paradox is urgently required to turn the health potential of nutritional approaches into actual health benefits. For this purpose, it is first and foremost required to define the specific domain of nutritional approaches as opposed to straightforward transposition of the randomized controlled trial methodology from clinical pharmacology to nutrition interventions.

## 5. From Single Nutrients to Dietary Patterns

Approaches that reduce the link between nutrition and health or diseases to single macro- or micro-nutrient relationships actually started with the discovery of the role of vitamins. However, applying this principle over the years, by using the epidemiological approach controlling for residual confounding factors as well as by applying the pharmacological approach of randomized controlled trials (RCT), has left the scientific community and the general public in great confusion about what is good or bad for health. Especially research on the optimal macronutrient composition has shown that this approach is not feasible since reducing one macronutrient automatically leads to a relative increase of the others given an isoenergetic diet. It is important to realize that in real life, nutritional factors are not isolated factors/actors on health status, but rather a proxy for nutritional habits that include many more other relevant nutrients as well [24]. Accordingly, nutritional interventions that focus on a single factor may not be specific, and affect intake of other macro- and micro-nutrients as well, and thus lead to unexpected outcomes. Additionally, interactions between different nutrients and/or interactions with concomitant pharmacological interventions may exert prominent health effects that can either be considered a “confounder”, or the other way round, be used to optimize benefit.

Mapping of nutritional profiles and their relation to health status, rather than assessing single nutrients, can guide a way forward and pave the way for rational interventions in nutritional habits. This requires reliable assessment of multiple nutrients simultaneously, and assessment of clustering and interaction patterns. This should preferably be linked to data on intake of food products, physical activity, and data on health status. Detected patterns should be validated by independent confirmation in a different population and/or by intervention data that serve as a perturbation of the system [25].

Obviously, the infrastructural requirements for such an approach are not trivial. Very large populations are required, with detailed characterization of multiple relevant nutritional factors to enable valid pattern recognition, along with a marker profile of nutritional status and multi-level health characteristics. Objective, accurate assessment of nutritional exposure (rather than estimates of intake) is a strategic advantage to this purpose. Cross-validation with intervention studies can serve to support the robustness of findings. For example, the LifeLines general population cohort in the Northern Netherlands and its embedding in the clinical setting of the University Medical Center Groningen, with ample opportunities for alignment and cross-validation with clinical interventions, provides a unique infrastructure for innovative nutritional profiling. Hence, LifeLines can provide substantial impetus to the design of rational multifactorial nutrition interventions, with the potential of exerting health benefits in daily life [26].

Evidence from both observational and intervention studies shows that individuals with dietary patterns more like a Mediterranean Diet (MD) live longer and have reduced risk of a wide range of age-related diseases including cardiovascular disease, dementia, and cancers [27,28,29,30,31]. In the LiveWell Programme, a suite of pragmatic interventions is being developed and piloted to change key behaviors and, in particular, to help shift eating patterns towards a more MD-like dietary pattern [32]. The intervention is targeted at people within the retirement transition which is considered as a possible key window of opportunity to establish new behavioural habits that will promote health and wellbeing in later life [33]. In addition, to make the intervention stratified (or personalized), scalable and sustainable, the intervention is delivered digitally. LiveWell targets three lifestyle domains: physical activity, dietary patterns, and social relationships. Heretofore, evidence on effective intervention components in each domain has been accumulated. The next phase is to integrate the evidence from this research, and, through a process of co-design involving stakeholders (such as those who would receive, deliver and purchase the interventions), produce a prototype “suite” of interventions [32].

Aging is a highly complex process and individual aging trajectories are heterogeneous [34]. However, there is no gold standard for assessing healthy aging and this creates difficulties when conducting and comparing research on aging across studies. The concept of the “Healthy Aging Phenotype” (HAP) was adopted and used to (1) identify the most important features of the HAP and (2) identify/develop tools for measurement of those features [35]. A tentative panel of (bio)markers of physiological and metabolic health, physical capability, cognitive function, social wellbeing, and psychological wellbeing was selected which may be useful in characterizing the HAP and which may have utility as outcome measures in intervention studies. In addition, a number of tools have been identified which could be applied in community-based intervention studies designed to enhance healthy aging [36].

## 6. Nutritional Assessment and Needs in Patients

There is convincing evidence that nutritional depletion adversely affects disease progression and outcome of surgical and medical interventions in patients suffering from acute or chronic disease [37]. Furthermore, it is well documented that the prevalence of nutritional depletion is high in various patient groups and increasing due to an aging society [38]. Nevertheless, nutritional assessment and intervention are still not incorporated into standard medical practice.

As discussed above, first of all it is important to clearly define nutritional status and reach consensus about screening and assessment tools [39,40]. Furthermore, nutritional needs should not only be identified from a body weight and energy balance perspective but also consider the impact of body composition abnormalities and nutrient deficiencies on clinical outcome [41]. It is important to position nutritional management as an integrated part of patient care and to acknowledge that management of nutritional depletion requires a multidisciplinary approach [42]. Guidelines by nutritional societies are useful, but being part of clinical consensus statements and guidelines from the respective medical societies is crucial. More research is needed to prove efficacy of nutritional interventions in specific patient groups. Advantages of targeted nutritional intervention studies in patients is that clear “hard” endpoints can be identified and measured, but future studies should focus on better identifying and phenotyping patients at risk as well as determining the “window of opportunity” for nutritional intervention in the disease trajectory. Since dietary habits form an integral component of someone’s lifestyle, active involvement of the patient is crucial for successful nutritional intervention and maintenance. Innovative strategies including psychological interventions and information and communications technology (ICT)-based guidance as well as counselling and shared decision making are yet poorly explored but recommended to improve efficacy and compliance. Furthermore, there is a clear need for more cost-effectiveness studies of nutritional counselling and supplementation to support decision making about reimbursement of these interventions. Next to the conventional approach of designing randomized clinical trials, cost-effectiveness modelling studies are proposed to simulate potential long-term effects of changes in weight and body composition on health status, healthcare utilization, and costs. The latter is necessary because the costs of nutritional intervention in less advanced chronic disease and in promoting active aging are likely to precede the benefits by far.

## 7. Conclusions and Recommendations

To increase awareness of malnutrition and its consequences in the community, schools, care homes, and hospitals should form a fundamental part of the strategy to fight malnutrition in the general population and especially in at-risk groups, including the elderly. Routine screening for malnutrition and low status of essential micronutrients across health and social services should start with such risk groups, e.g., pregnant women, infants and children, the elderly, and hospitalized patients. Encouragement of healthy diets and improvement of nutritious food intake should be a key part of care planning to prevent malnutrition and inadequate micronutrient status. Even though undoubtedly a balanced diet is the best way of achieving sufficient intake of nutrients, it may be difficult for individuals to get the required amount of nutrients due to poor energy intake or social and health issues, putting them at risk of nutrient inadequacies.

In light of the antecedents, the following points should be considered by the responsible stakeholders:

– There is an urgent need of strategies aimed at communicating optimal nutrition in order to create awareness of malnutrition amongst policymakers, the general public, and health and social care professionals. Targeted dissemination of respective information is recommended.
– Strategies aimed at preventing malnutrition require early identification of the condition. This needs active surveillance of nutrient status using validated biomarkers in at risk populations on admission to health and social services in order to take timely action and prevent the individual’s malnutrition as well as the high economic costs of inaction.
– Strategies aimed at preventing malnutrition require high quality evidence about the effect of interventions on longer-term outcomes. Prioritization towards nutrition research is needed.
– Strategies aimed at preventing malnutrition require periodic evaluations of progress and reporting of actions needed to address the progress of targeted individuals. Centrally stored data of nutritional status could assist in identifying the public health issues related to malnutrition in risk groups.
– Action implementation should be based on nutrition surveys, existing evidence of nutrition interventions, and recommendations and guidelines by (inter)national organizations and professionals.
– Strategies aimed at preventing malnutrition require capacity for trained caregivers and health professionals to identify and treat malnutrition. This requires that governments invest in resources that enable screening and understanding effective interventions that address malnutrition in the general population.

Together with other socioeconomic issues, nutrition should be considered as an integral part of the solution to achieve nutrient adequacy and support healthy aging. Nutrient interventions could considerably contribute to cost-effective interventions. Despite the fact that more evidence is needed, implementation should start now.
